# *Mycoplasma hyorhinis* Activates the NLRP3 Inflammasome and Promotes Migration and Invasion of Gastric Cancer Cells

**DOI:** 10.1371/journal.pone.0077955

**Published:** 2013-11-06

**Authors:** Yongfen Xu, Hua Li, Wei Chen, Xiaomin Yao, Yue Xing, Xun Wang, Jin Zhong, Guangxun Meng

**Affiliations:** 1 Key Laboratory of Molecular Virology and Immunology, Institut Pasteur of Shanghai, Shanghai Institutes for Biological Sciences, Chinese Academy of Sciences, Shanghai, China; 2 Shanghai Blood Center, Shanghai, China; University of Hyderabad, India

## Abstract

**Background:**

*Mycoplasma hyorhinis* (*M.hyorhinis, M.hy*) is associated with development of gastric and prostate cancers. The NLRP3 inflammasome, a protein complex controlling maturation of important pro-inflammatory cytokines interleukin (IL)-1β and IL-18, is also involved in tumorigenesis and metastasis of various cancers.

**Methodology/Principal Findings:**

To clarify whether *M.hy* promoted tumor development via inflammasome activation, we analyzed monocytes for IL-1β and IL-18 production upon *M.hy* challenge. When exposed to *M.hy*, human monocytes exhibited rapid and robust IL-1β and IL-18 secretion. We further identified that lipid-associated membrane protein (LAMP) from *M.hy* was responsible for IL-1β induction. Applying competitive inhibitors, gene specific shRNA and gene targeted mice, we verified that *M.hy* induced IL-1β secretion was NLRP3-dependent *in vitro* and *in vivo*. Cathepsin B activity, K^+^ efflux, Ca^2+^ influx and ROS production were all required for the NLRP3 inflammasome activation by *M.hy*. Importantly, it is IL-1β but not IL-18 produced from macrophages challenged with *M.hy* promoted gastric cancer cell migration and invasion.

**Conclusions:**

Our data suggest that activation of the NLRP3 inflammasome by *M.hy* may be associated with its promotion of gastric cancer metastasis, and anti-*M.hy* therapy or limiting NLRP3 signaling could be effective approach for control of gastric cancer progress.

## Introduction

Mycoplasmas are pleomorphic, wall free, prokaryotic organisms that reside either on the eukaryotic cell membranes or inside the cells, and they are the smallest organisms capable of self replication [1]. To date, at least 16 mycoplasma species have been isolated from humans [2]. *Mycoplasma hyorhinis* (*M.hy*) was considered non-pathogenic to humans as it usually infects swine leading to respiratory tract disease and inflammation of the chest and joints [3], [4]. However, accumulating evidence suggests that *M.hy* infection in humans does result in clinical outcomes. *M.hy* was found in 56% of gastric carcinoma, 55% of colon carcinoma and 52.6% of lung carcinoma biopsies [5]. Moreover, 36% men with benign prostatic hyperplasia (BPH) and 52% men with prostate cancer are *M.hy* sero-positive. These clinical findings suggest a possible connection between *M.hy* exposure with gastric, colon, lung and prostate cancers [5], [6].

Upon microbial infection, host pattern recognition receptors (PRRs) such as TLRs sense the pathogens and trigger the synthesis of pro-inflammatory cytokines such as pro-IL-1β and pro-IL-18 via NF-κB activation. At the same time, another group of PRRs including NLRP3 recruit the adaptor protein ASC and lead to the activation of caspase-1, which is an active protease that cleaves the precursor form of cytokines including pro-IL-1β and pro-IL-18 into mature, secreted form [7]. Pathogens or challenging agents cause potassium efflux, mitochondria damage, mitochondria DNA release, ROS production, intracellular calcium increase or cellular cyclic AMP decrease in innate immune cells, which are all involved in caspase-1 activation [8], [9], [10], [11], [12], [13]. During this process, the NLRP3, ASC and pro-caspase-1 form a molecular platform called inflammasome. So far, a number of inflammasomes have been identified, of them, the NLRP3 inflammasome has been found associated with tumor development [14], [15], although controversy exists from different models [16], [17]. Nonetheless, IL-1β was reported to promote tumor cell growth and metastasis by inducing several pro-metastatic genes such as matrix metalloproteinases and endothelial adhesion molecules, as well as TGF-β, chemokines and growth factors [18]. In a Korean population, the combination of increased mucosal IL-1β level and homozygosity for IL-1β -31T single nucleotide polymorphism (SNP) are both associated with increased risk for gastric cancer [18]. Furthermore, Tu et al. found that stomach-specific expression of human IL-1β in transgenic mice led to spontaneous gastric inflammation and cancer [19], further suggesting that IL-1β may promote human gastric carcinogenesis. In contrast, IL-18 enhances NK cell activity, reduces tumorigenesis, induces apoptosis and inhibits angiogenesis in tumor cells to exert anti-tumor effects [20], [21]. In addition, an inappropriate production of IL-18 was found to contribute to the pathogenesis of cancers and may influence the clinical outcome of patients [22]. IL-18 was reported to stimulate matrix metalloprotease-9 production, resulting in increased migration and invasion in coronary artery smooth muscle cells and HL-60 myeloid leukemia cells [23], [24]. It was also reported that the serum IL-18 level in gastric cancer patient group was significantly higher than that in gastric ulcer patient group [25] and IL-18 can increase metastasis and immune escape of stomach cancer via the down-regulation of CD70 and maintenance of CD44 in human gastric cancer cell line NCI-N87 and SNU16 [26]. It is also a critical mediator of VEGF-enhanced migration in human gastric cancer cell lines SNU-601 [27]. The above findings suggest that *Mycoplasma hyorhinis* may promote migration and invasion of gastric cancer cells by activating inflammasome.

To date, a wide spectrum of microbes including viruses, bacteria, fungi and protozoa have been identified to activate the NLRP3 inflammasome [28]. A recent study showed that *Mycoplasma pneumoniae* was also able to induce IL-1β production in human cells [29]. However, whether *M.hy*, which is an important factor in gastric cancer development as mentioned above [5], [30], [31], activates the NLRP3 inflammasome and whether this activation contributes to the gastric cancer development remain unknown. In this study, we found that *M.hy* triggered IL-1β secretion in a NLRP3 inflammasome-dependent manner, and the resulting IL-1β induced migration and invasion of gastric cancer cells.

## Results

### *M.hy* Triggers IL-1β and IL-18 Production in THP-1 Cells

**Figure 1:**
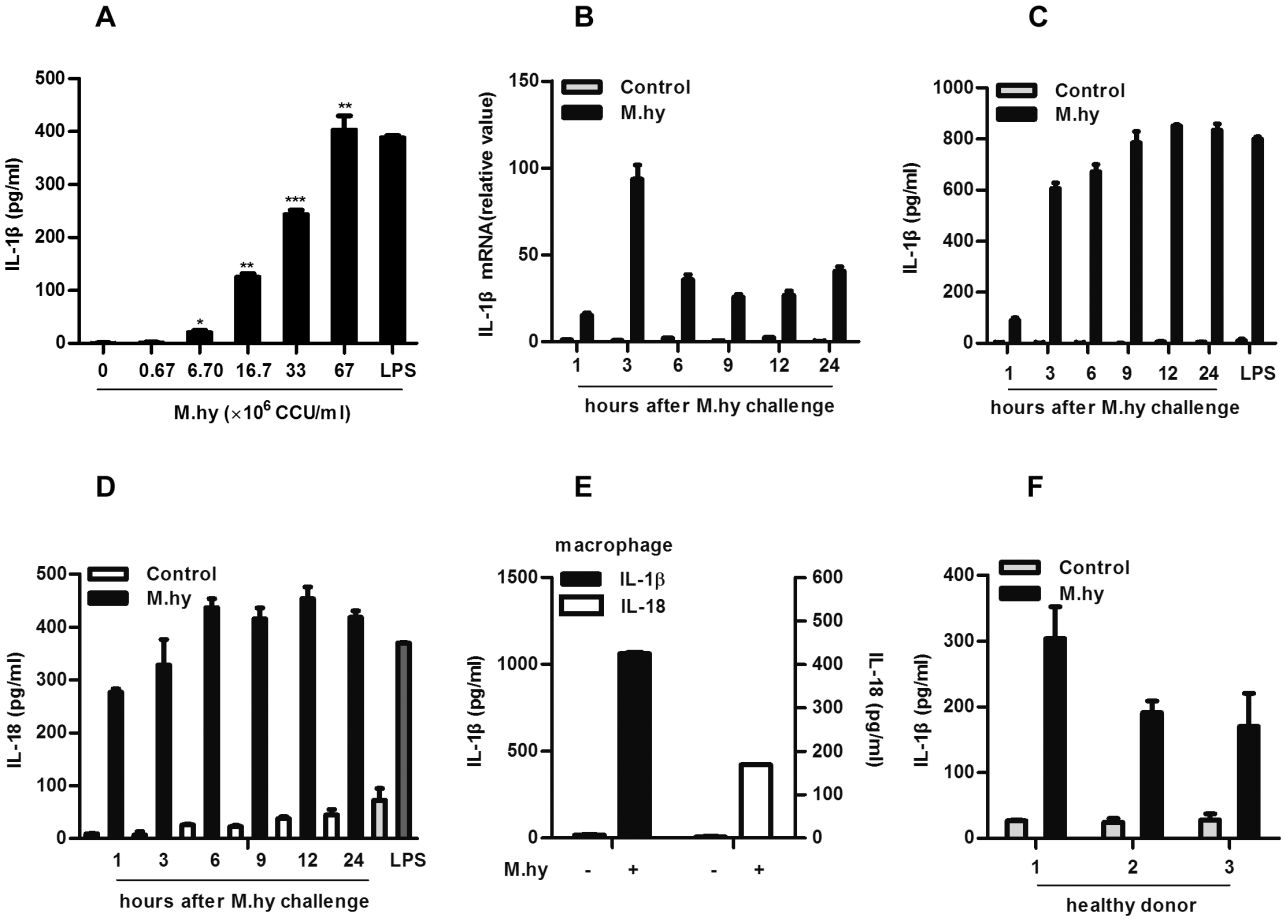
*M.hy* triggers IL-1β and IL-18 production in human monocytic cells. A, 5×10^4^ THP-1 cells were treated with *M.hy* at different doses, 12 hours later the supernatants were harvested for IL-1β ELISA. B-D, 2×10^5^ THP-1 cells were treated with *M.hy* at a concentration of 6.7×10^7^ CCU/ml, cells and supernatants were collected at different time points for IL-1β mRNA (B) and IL-1β (C) as well as IL-18 protein (D) detection by real time-PCR and ELISA as indicated. E–F, 5×10^4^ PMA-induced macrophages (E) and human primary monocytes (F) were treated with *M.hy*, 12 hours later the supernatants were harvested for cytokines ELISA. The values represent the mean ± the standard deviation (SD) of three independent experiments. ***represents P<0.001, **represents P<0.01 and *represents P<0.05 in comparison with control in statistic analysis.

To determine whether *M.hy* induces IL-1β production from innate immune cells, we monitored mature IL-1β levels in human monocytic cell line THP-1 cells challenged with different amounts of *M.hy*. In this experiment, a strong induction of IL-1β in a dose-dependent manner was observed (Figure 1A). Next, we measured the pro-IL-1β mRNA levels in the cells and mature IL-1β protein levels in the culture supernatants at different time points after *M.hy* challenge. It was observed that IL-1β mRNA levels peaked at 3 hours after challenge (Figure 1B) and mature IL-1β (Figure 1C) and IL-18 (Figure 1D) protein levels peaked at 12 hours after challenge. Moreover, THP-1 derived macrophages also exhibited robust secretion of IL-1β, IL-18 as well as other inflammatory cytokines such as IL-6 and IL-8 (Figure 1E, Figure S1). To confirm the above findings in THP-1 cell line, we examined the IL-1β production in primary human monocytes from healthy donors challenged with *M.hy*, wherein IL-1β was also strongly induced (Figure 1F). These data indicated that *M.hy* triggered robust IL-1β and IL-18 secretion from human myeloid cells.

### LAMP derived from *M.hy* is Responsible for IL-1β Induction through TLR2

**Figure 2:**
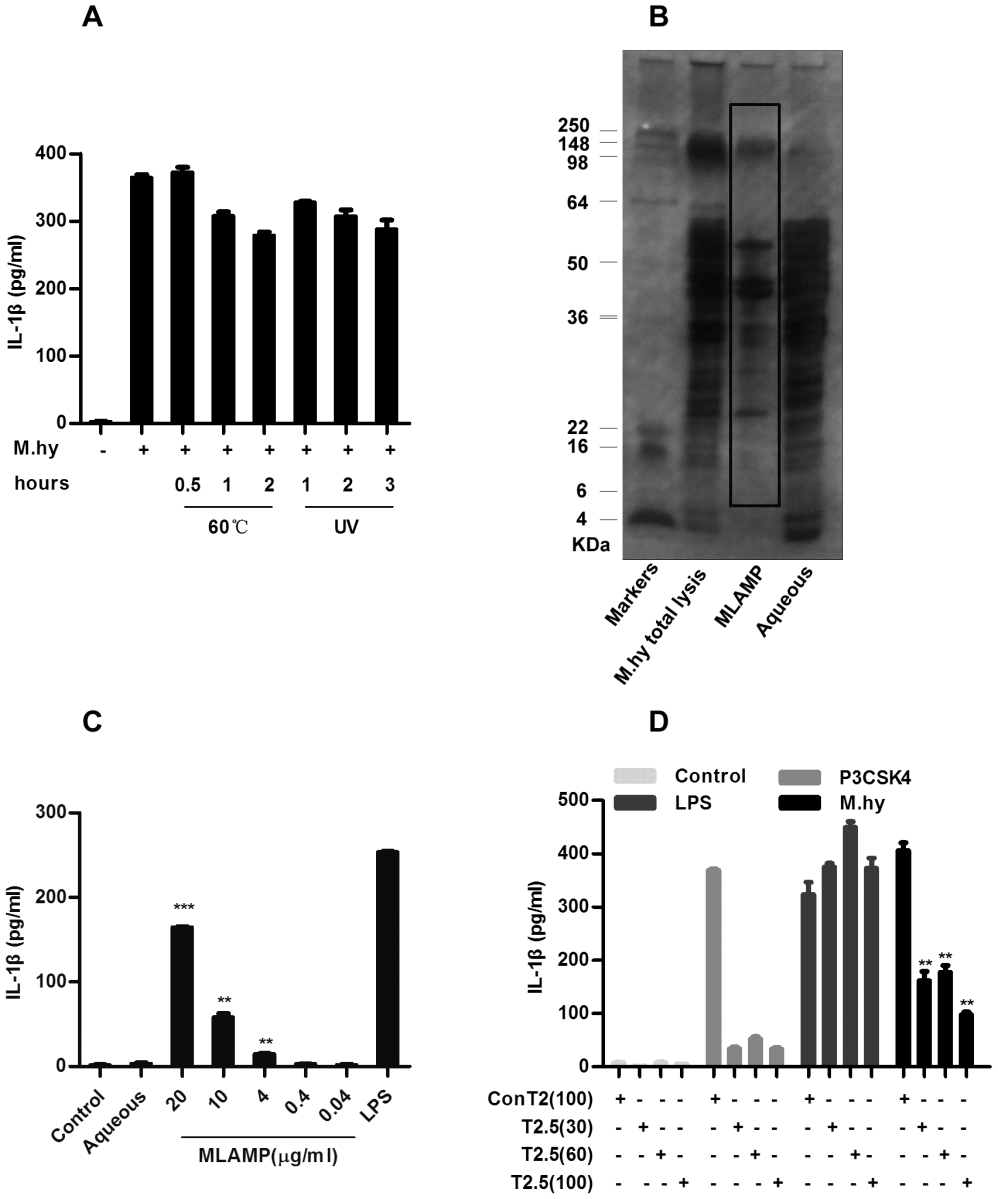
MLAMP is responsible for IL-1β induction through TLR2. A, 5×10^4^ THP-1 cells were treated with heat or ultra-violet irradiation inactivated *M.hy* for 6 hours and the supernatants were harvested for IL-1β ELISA. B, Total protein of *M.hy*, MLAMP extracted from *M.hy* and the aqueous protein were detected by SDS-PAGE and silver staining. C, 5×10^4^ THP-1 cells were treated with MLAMP at different concentrations and aqueous, 6 hours later the supernatants were harvested for IL-1β ELISA. D, 5×10^4^ THP-1 cells were pre-incubated with the indicated amounts of mAb T2.5 or conT2 (µg/ml) for 0.5 hour and challenged with TLR2 ligand P3CSK4 (positive control), TLR4 ligand LPS (negative control) and *M.hy* at a concentration of 6.7×10^7^ CCU/ml subsequently for 6 hours, the supernatants were harvested for IL-1β ELISA. The values represent the mean ± the standard deviation of three independent experiments. ***represents P<0.001, **represents P<0.01 and *represents P<0.05 in comparison with control in statistic analysis.

Next we investigated whether replication of *M.hy* was required for IL-1β production in monocytes. As shown in Figure 2A, *M* *.hy* inactivated by heating or ultra-violet (UV) treatment induced as much IL-1β secretion from THP-1 cells as live *M.hy*. This indicated that certain heat- and UV- resistant component of *M.hy* was responsible for induction of IL-1β secretion. Lipid-associated membrane protein (LAMP) is abundantly expressed on the surface of *M.hy* [32], and previous study found that membrane lipoproteins from other mycoplasma species induced IL-1β secretion [33]. We thus speculated that *M.hy* derived LAMP (MLAMP) may be responsible for the IL-1β induction in THP-1 cells. We then extracted LAMP from *M.hy* (Figure 2B) and tested the capability of MLAMP to induce IL-1β secretion in THP-1 cells. We found that MLAMP clearly induced secretion of IL-1β in a dose-dependent manner, while the aqueous phase of *M.hy* extract was not able to do so (Figure 2C). To exclude the possible RNA and/or DNA contamination in MLAMP, we treated the MLAPMs with RNase and DNase and found that MLAMP was the major component for IL-1β induction (Figure S2).

It was reported that MLAMP mainly activated NF-κB through TLR2 and TLR6 [34], [35]. Therefore, we first applied a TLR2 neutralizing antibody T2.5 to block the TLR2 signal, with ConT2 serving as an isotype control [36]. As shown in Figure 2D, T2.5 completely blocked the IL-1β secretion stimulated by TLR2 agonist P3CSK4 but not by the TLR4 ligand LPS. Importantly, IL-1β secretion from *M.hy* infected cells was strongly reduced by T2.5, indicating that TLR2 was involved in MLAMP triggered IL-1β secretion in human monocytes. Interestingly, IL-1β secretion from *M.hy* infected cells was not completely blocked by T2.5 (Figure 2D), which suggested that TLR2-independent signaling pathway was also involved in MLAMP triggered IL-1β secretion, which deserves further investigation in future.

### *M.hy* Induces IL-1β Secretion through Activation of the NLRP3 Inflammasome

**Figure 3:**
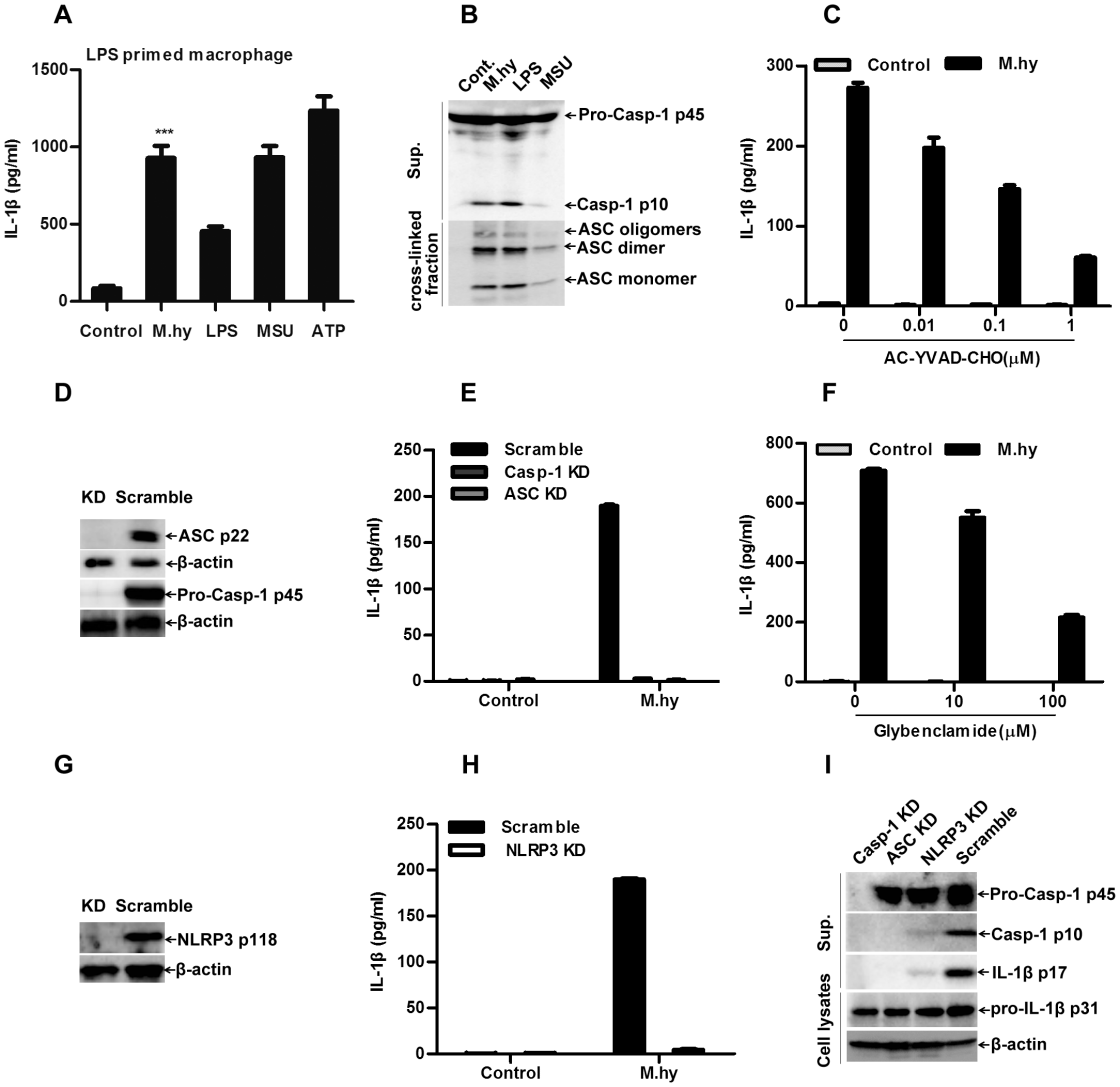
*M.hy* induced IL-1β production is NLRP3 inflammasome dependent. A, 1×10^5^ PMA-induced macrophages were primed with 50 ng/ml LPS, then treated with *M.hy*, LPS, MSU or ATP for 5 hours, the supernatants were harvested for IL-1β ELISA. B, The cleavage of caspase-1 and oligomerization of ASC in the inflammasome-enriched and cross-linked lysates of THP-1 derived macrophages treated with *M.hy*, LPS and MSU for 6 hours. Monomers, dimers, and oligomers of ASC were indicated accordingly. C, 5×10^4^ THP-1 were pretreated with caspase-1 inhibitor AC-YVAD-CHO and then challenged with *M.hy* at a concentration of 6.7×10^7^ CCU/ml, 12 hours later the supernatants were harvested for IL-1β ELISA. D, Successful silencing (knock-down, KD) of ASC and caspase-1 was verified by western blotting. E, 5×10^4^ THP-1 scramble, caspase-1 KD cells and ASC KD cells were treated with *M.hy* at a concentration of 6.7×10^7^ CCU/ml, 12 hours later the supernatants were collected for IL-1β ELISA. F, 5×10^4^ THP-1 cells were pretreated with NLRP3 inflammasome inhibitor Glybenclamide and then challenged with *M.hy*, 12 hours later the supernatants were harvested for IL-1β ELISA. G, Successful silencing of NLRP3 was verified by western blotting. H, 5×10^4^ THP-1 scramble and NLRP3 KD cells were treated with *M.hy* at a concentration of 6.7×10^7^ CCU/ml, 12 hours later the supernatants were collected for IL-1β ELISA. I, Immunoblotting analysis of pro-Caspase-1 and mature (P10) form of caspase-1 and mature IL-1β (P17) in supernatants and pro-IL-1β (P31) and β-actin in cell extracts of THP-1 scramble and caspase-1 KD, ASC KD and NLRP3 KD cells treated with *M.hy* for 6 hours. Data presented are mean ± SD of one representative out of three independent experiments. ***represents P<0.001 in comparison with control in statistic analysis.

To test whether *M.hy* induced IL-1β secretion through inflammasome activation, we first utilized LPS primed THP-1 derived macrophages to check if *M.hy* can activate inflammasome like ATP or MSU, which are known inflammasome activators. As shown in Figure 3A, *M* *.hy* was able to induce IL-1β release in the primed cells. Next we examined the cleavage of caspase-1 and oligomerization of ASC, two important makers for the inflammasome activation. We found that *M.hy* promoted the cleavage of caspase-1 and oligomerization of ASC (Figure 3B), indicating a direct activation of inflammasome by *M.hy.*

Furthermore, we tested whether *M.hy* induced IL-1β secretion was dependent on certain inflammasome components. First, we found that the specific caspase-1 inhibitor AC-YVAD-CHO decreased IL-1β secretion in THP-1 cells in a dose-dependent manner (Figure 3C). Next, when specific shRNA was employed to silence the expression of caspase-1 and ASC (Figure 3D), IL-1β production was strongly decreased upon *M.hy* challenge (Figure 3E), indicating that *M.hy* induced IL-1β secretion was dependent on caspase-1 and ASC.

Then, we tested whether NLRP3 was required for *M.hy* induced IL-1β secretion. First, we found that the NLRP3 inhibitor Glybenclamide suppressed *M.hy* induced IL-1β secretion in a dose-dependent manner (Figure 3F) [37]. When specific shRNA was employed to silence the expression of NLRP3 (Figure 3G), IL-1β production was strongly decreased upon *M.hy* challenge (Figure 3H), indicating that *M.hy* induced IL-1β secretion was dependent on NLRP3. This was further confirmed by immunoblotting, in which the caspase-1 activation and IL-1β production were both NLRP3 dependent (Figure 3I). Moreover, we further found that MLAMP was the major component responsible for NLRP3 inflammasome activation by *M.hy* (Figure S2C). In addition, AIM2 inflammasome was reported to be activated by DNA [38], and we found that *M.hy* DNA induced IL-1β secretion via AIM2 inflammasome (Figure S3A). Interestingly, *M.hy* induced IL-1β secretion was mainly dependent on NLRP3, while AIM2 was only partially involved (Figure S3A). Taken together, these results clearly demonstrated that *M.hy* induced IL-1β secretion through activation of the NLRP3 inflammasome in human monocytic cells.

### Mechanisms Underlying *M.hy* Triggered NLRP3 Inflammasome Activation

**Figure 4:**
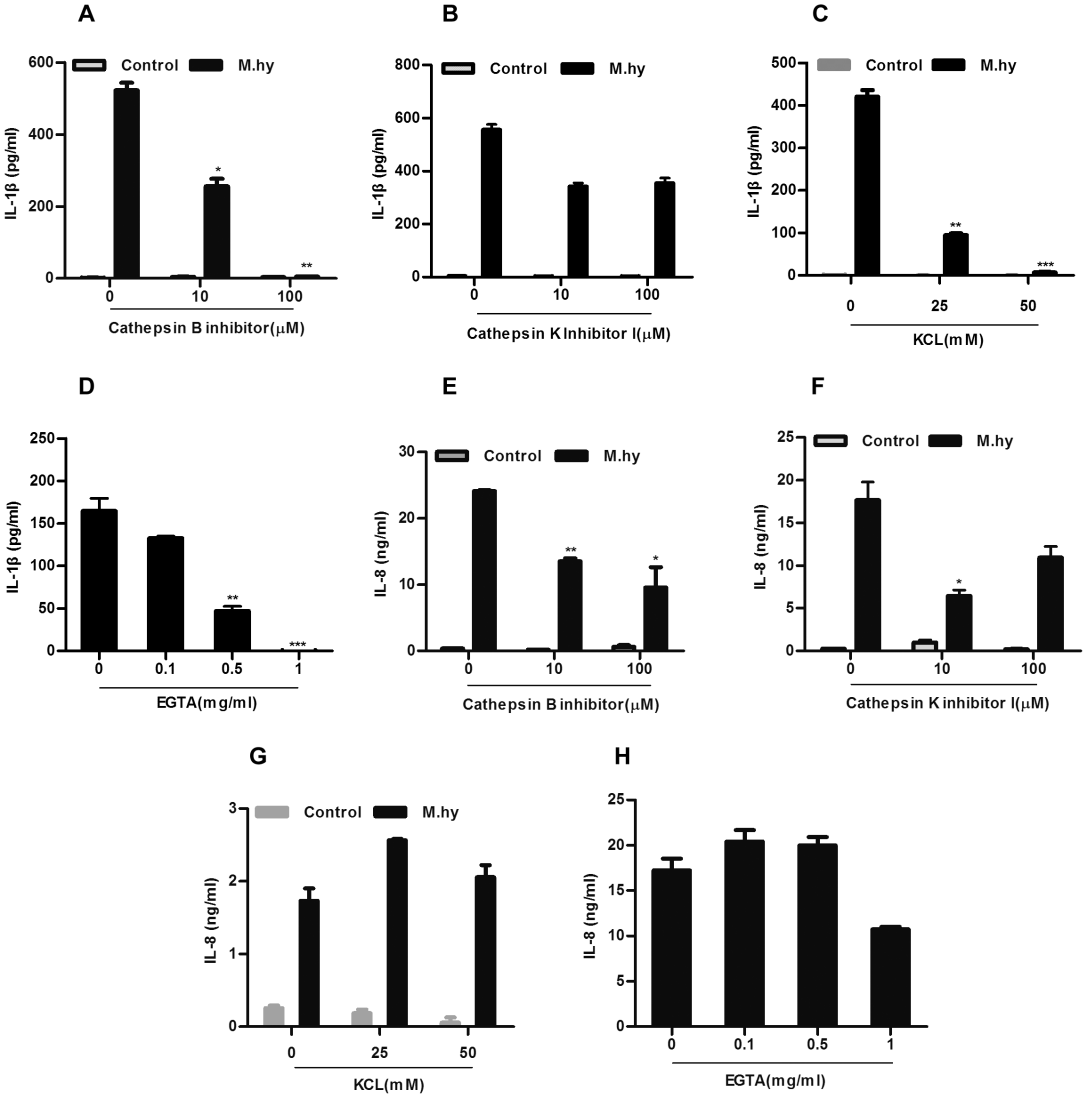
Mechanism of NLRP3 inflammasome activation in response to *M.hy* challenge. 5×10^4^ THP-1 were pretreated with different doses of Cathepsin B inhibitor CA-074 Me (A, E) or Cathepsin K Inhibitor I (B, F), KCL (C,G) or EGTA(D, H), then were challenged with *M.hy* at a concentration of 6.7×10^7^ CCU/ml, 6 hours later the supernatants were harvested for IL-8 and IL-1β ELISA at the same time. Data presented are mean ± SD of one representative out of three independent experiments. ***represents P<0.001, **represents P<0.01 and *represents P<0.05 in comparison with control in statistic analysis.

Activation of NLRP3 inflammasome by a variety of stimuli depends on Cathepsin B activity, K^+^ efflux, calcium influx and/or production of reactive oxygen species (ROS) [11], [12], [13], [39]. To explore the mechanisms underlying IL-1β release in response to *M.hy* challenge, we first applied Cathepsin B-specific inhibitor CA-074 Me and observed a significant attenuation of IL-1β secretion upon *M.hy* challenge. At a concentration of 100 µM, CA-074 Me completely blocked IL-1β release (Figure 4A). This inhibition was not due to any toxic effect to the cells as IL-8 secretion affected by this inhibitor was very mild (Figure 4E). Importantly, when the cells were treated with Cathepsin K inhibitor I, the decrease of IL-1β secretion was not evident at all (Figure 4B and 4F), suggesting that the Cathepsin B activity was specifically involved in NLRP3 inflammasome activation during *M.hy* challenge. In addition, when we blocked K^+^ efflux by increasing the extracellular K^+^ concentration, IL-1β secretion by THP-1 cells upon *M.hy* challenge was significantly reduced in a dose-dependent manner (Figure 4C). Again, there was no toxic effect from KCL as IL-8 production from the same cells was normal (Figure 4G). To figure out whether calcium influx helped inflammasome activation, we added different doses of EGTA in the cell culture medium and found that EGTA treatment blocked *M.hy*-induced IL-1β secretion in a dose-dependent manner, and no toxic effect from EGTA was observed as evidenced by IL-8 production (Figure 4D and 4H).

**Figure 5:**
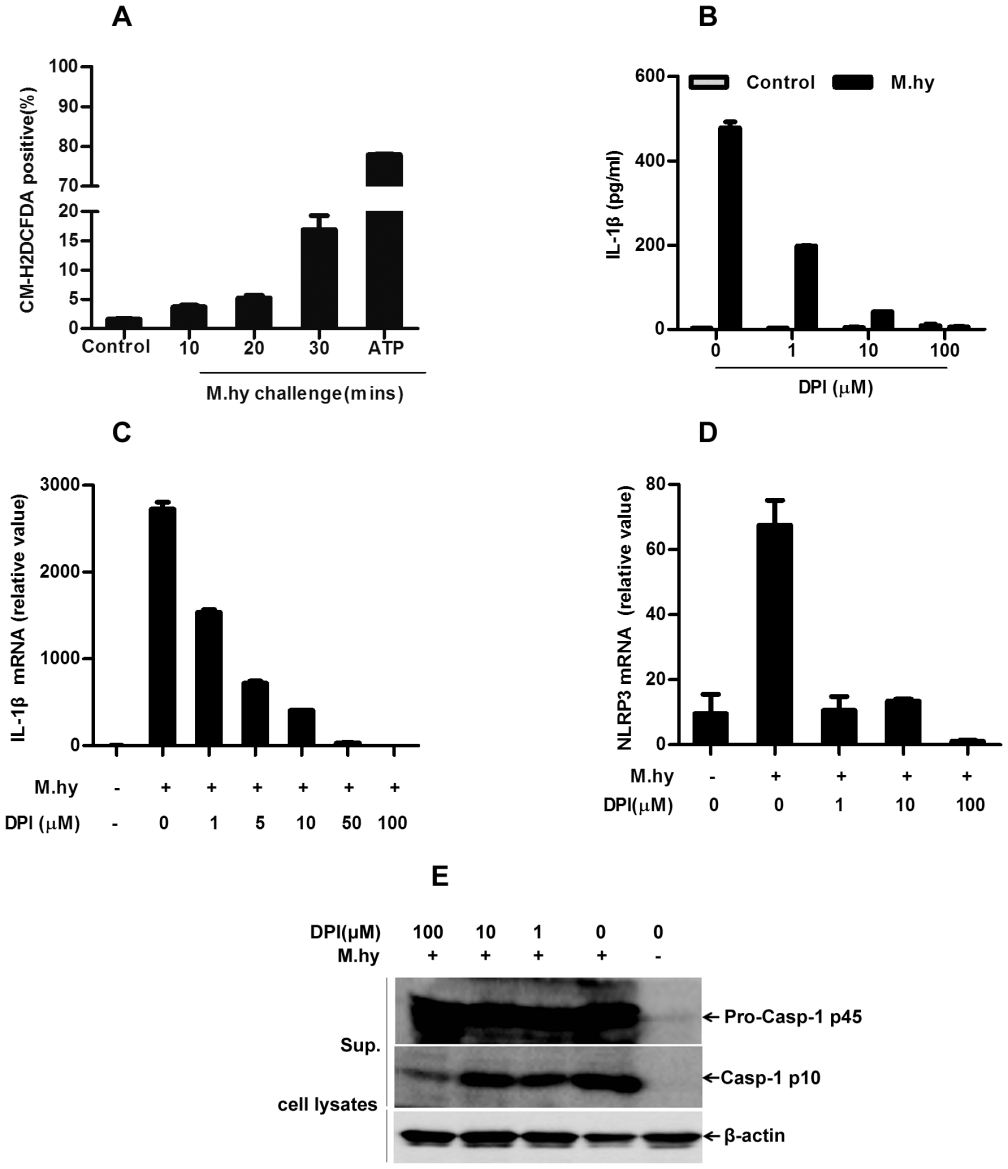
NLRP3 inflammasome activation in response to *M. hy* is dependent on ROS. A, Levels of ROS in *M.hy* treated THP-1 cells were analyzed by CM-H2DCFDA labeling. Data represents mean of the percentage of CM-H2DCFDA-positive cells. B, 5×10^4^ THP-1 cells were pre-incubated with the indicated amounts of DPI for 0.5 hour and challenged with *M.hy* subsequently for 6 hours, the supernatants were harvested for IL-1β ELISA. 1×10^5^ THP-1 cells (C) and PMA induced macrophages (D) were pre-incubated with the indicated amounts of DPI for 0.5 hour and challenged with *M.hy* (final concentration, 6.7×10^7^ CCU/ml) subsequently for 3 hours, the cells were lysed and mRNA were extracted for cDNA synthesis and finally for IL-1β and NLRP3 gene expression by real-time PCR. E, Immunoblot analysis of pro-caspase-1 and mature (P10) form of caspase-1 and mature IL-1β (P17) in supernatants and extracts of THP-1 derived macrophages treated with *M.hy* for 6 hours after DPI pretreatment for 0.5 hour. Data presented are mean ± SD of one representative out of three independent experiments.

Furthermore, we tested whether *M.hy* challenge induced ROS generation in THP-1 cells. For this assay, THP-1 cells were first loaded with the redox-sensitive fluorophore DCFDA and then challenged with *M.hy*, ATP was included as a positive control. As shown in Figure 5A, 16.9% CM-H2DCFDA positive cells were detected 30 minutes after *M.hy* treatment compared to 1.6% in untreated cells. These data suggested that ROS may contribute to NLRP3 inflammasome activation in response to *M.hy* challenge. To verify this possibility, we pretreated THP-1 cells with ROS inhibitor diphenyliodonium (DPI) for 30 minutes, and then challenged the cells with *M.hy* before measurement of IL-1β production 6 hours later. As expected, DPI dramatically reduced *M.hy* induced IL-1β release (Figure 5B). A recent study showed that ROS inhibitor such as DPI abolished IL-1β release in mouse macrophages by inhibiting NLRP3 gene expression [40]. Therefore, we also monitored the mRNA level of pro-IL-1β and NLRP3 under DPI treatment. We found that DPI markedly reduced the expression of pro-IL-1β and NLRP3 (Figure 5C–D), which was consistent with the finding from mouse cells [40]. In addition, we also examined the activation of caspase-1 and found that low doses (1 or 10 µM) of DPI treatment did not affect the caspase-1 activation while 100 µM of DPI inhibited caspase-1 activation (Figure 5E). This demonstrated that in human cells DPI interfered with NLRP3 inflammasome activation through inhibition of NLRP3 transcription as well as caspase-1 activity when high dose was applied. Collectively, these data suggest that Cathepsin B activity, K^+^ efflux, Ca^2+^ influx and ROS all contributed to NLRP3 inflammasome activation in response to *M.hy* challenge.

### *M.hy* Activates NLRP3 Inflammasome *in vivo*

**Figure 6:**
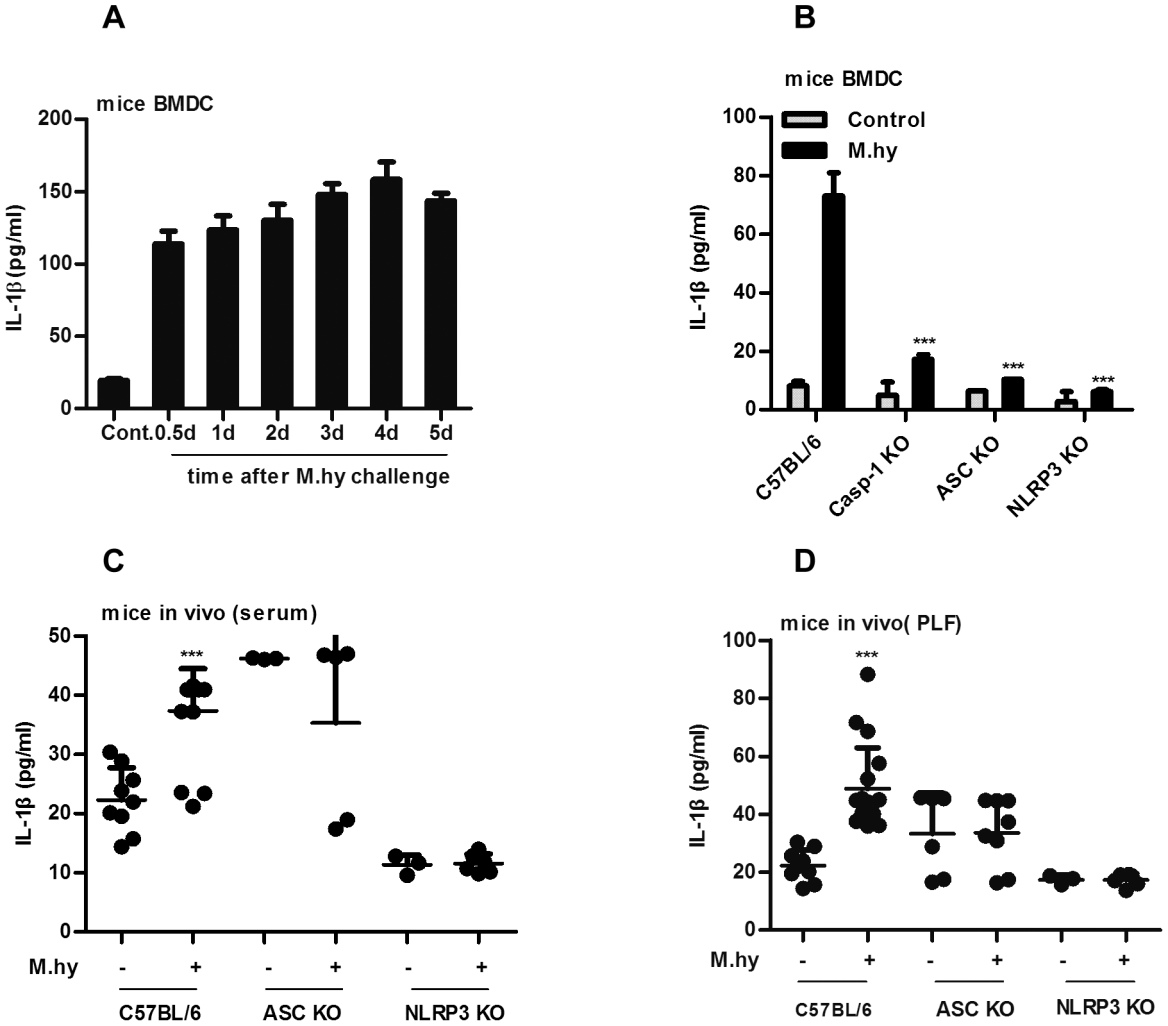
*M.hy* induces NLRP3 inflammasome activation in mouse BMDCs and *in vivo*. A, 1×10^5^ mouse bone marrow derived dendritic cells (BMDCs) were challenged with *M.hy* at a final concentration of 5×10^7^ CCU/ml at different time points, supernatants were harvested for IL-1β ELISA. B, 1×10^5^ BMDCs isolated from wild-type and caspase-1 deficient (knock-out, KO), ASC KO and NLRP3 KO mouse were challenged with *M.hy* at a final concentration of 5×10^7^ CCU/ml, 24 hours later the supernatants were harvested for IL-1β ELISA. C-D, C57BL/6, ASC KO and NLRP3 KO mice were injected i.p. with 5×10^8^ CCU/ml of *M.hy* in 200 µl of PBS. Mice were euthanized 6 hours after injection, and serum or peritoneal lavage fluid (PLF) were collected for IL-1β ELISA (C, D). The experiment was repeated three times, and the data were pooled and showed as mean ± SD. ***represents P<0.001 and **represents P<0.01 in comparison with control in statistic analysis.

When mouse bone marrow derived dendritic cells (BMDCs) were infected with *M.hy*, a sustained IL-1β induction was observed (Figure 6A). Further experiments on BMDCs isolated from wild-type (WT) mice or mice deficient for caspase-1, ASC or NLRP3 with *M.hy* challenge confirmed that *M.hy* induction of IL-1β was NLRP3 inflammasome dependent (Figure 6B). Furthermore, systemic inoculation of *M.hy* in WT mice induced IL-1β production in serum and peritoneal lavage fluid (PLF), but not in mice lacking ASC or NLRP3 (Figure 6C–D). Interestingly, the basal level of IL-1β in ASC deficient mice was clearly higher than that in WT or NLRP3 deficient mice, but challenge with *M.hy* did not further increase the secretion of IL-1β in such mice (Figure 6C–D). Collectively, these findings confirmed that *M.hy* also activated NLRP3 inflammasome in mice *in vitro* and *in vivo*.

### *M.hy* Induced IL-1β Promotes Gastric Tumor Cell Migration and Invasion

The above data clearly showed that *M.hy* was able to induce IL-1β production from innate immune cells through NLRP3 inflammasome activation. Early epidemic studies revealed a possible role of mycoplasma in the development of gastric cancer [5]. And *Mycoplasma hyorhinis* was demonstrated to promote tumor cell migration, invasion and metastasis *in vitro* and *in vivo* [41]. We hypothesized that there may exist another mechanism for this promotion, which is that *M.hy* may promote gastric carcinogenesis and/or metastasis by promoting IL-1β production. And we confirmed that *M.hy* promoted gastric cancer cell migration and invasion (Figure S5). Since *M.hy* was demonstrated to infect gastric cancer cells ([41], Figure S4), it is thus possible that *M.hy* may induce inflammasome activation in cancer cells directly. However, no inflammasome activation was detected in gastric cancer cells (Figure S6).

**Figure 7:**
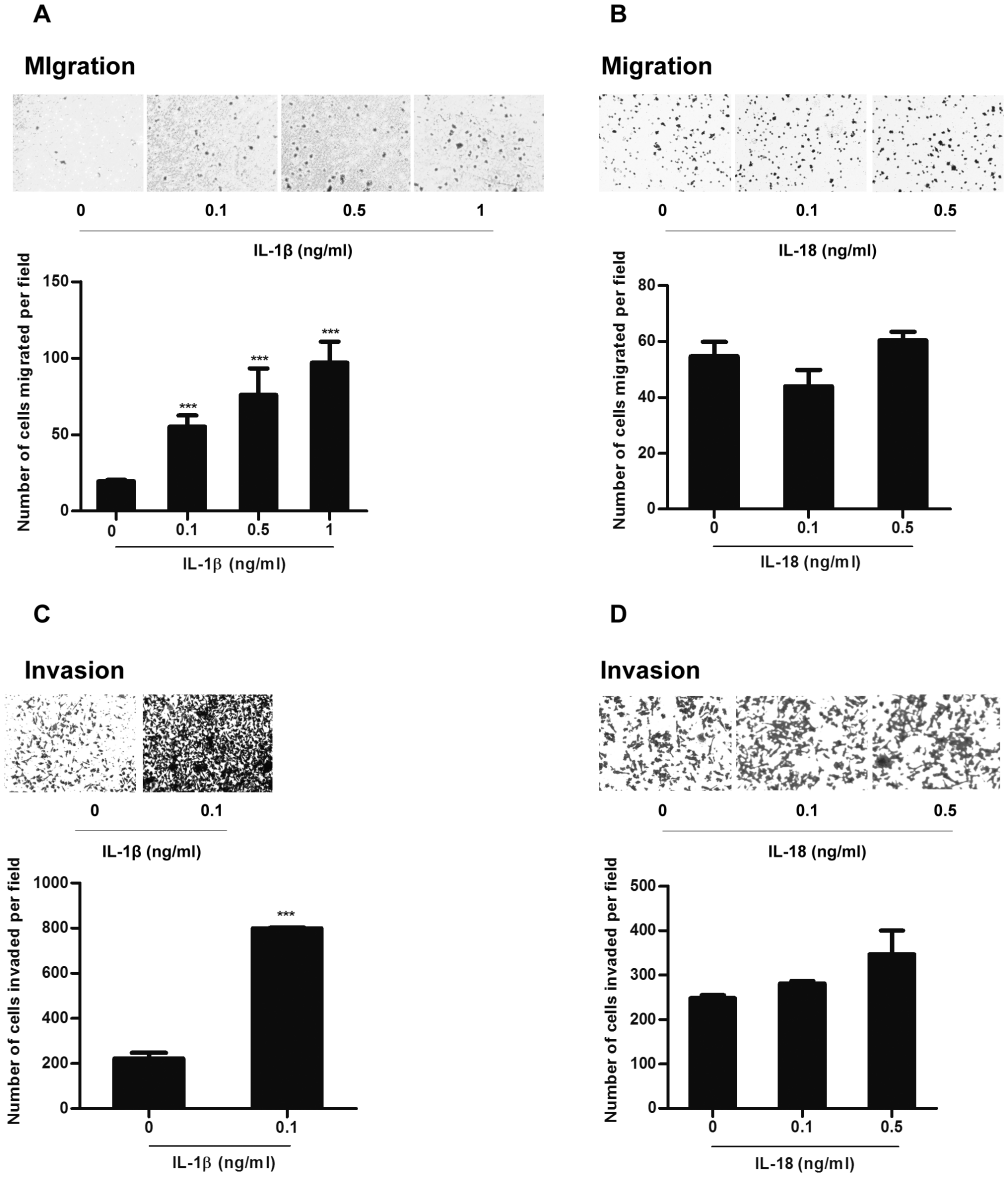
IL-1β but not IL-18 promotes gastric cancer cell migration and invasion. 5×10^4^ MGC-803 cells treated with indicated doses of IL-1β (A and C) or IL-18 (B and D) were analyzed by Transwell migration and invasion assays. The upper were migrated or invaded cells and the lower were average numbers of 4 microscopic fields for each corresponding experiment respectively. Data are representative of three independent experiments and error bars represent SD. ***represents P<0.001 in comparison with control in statistic analysis.

**Figure 8:**
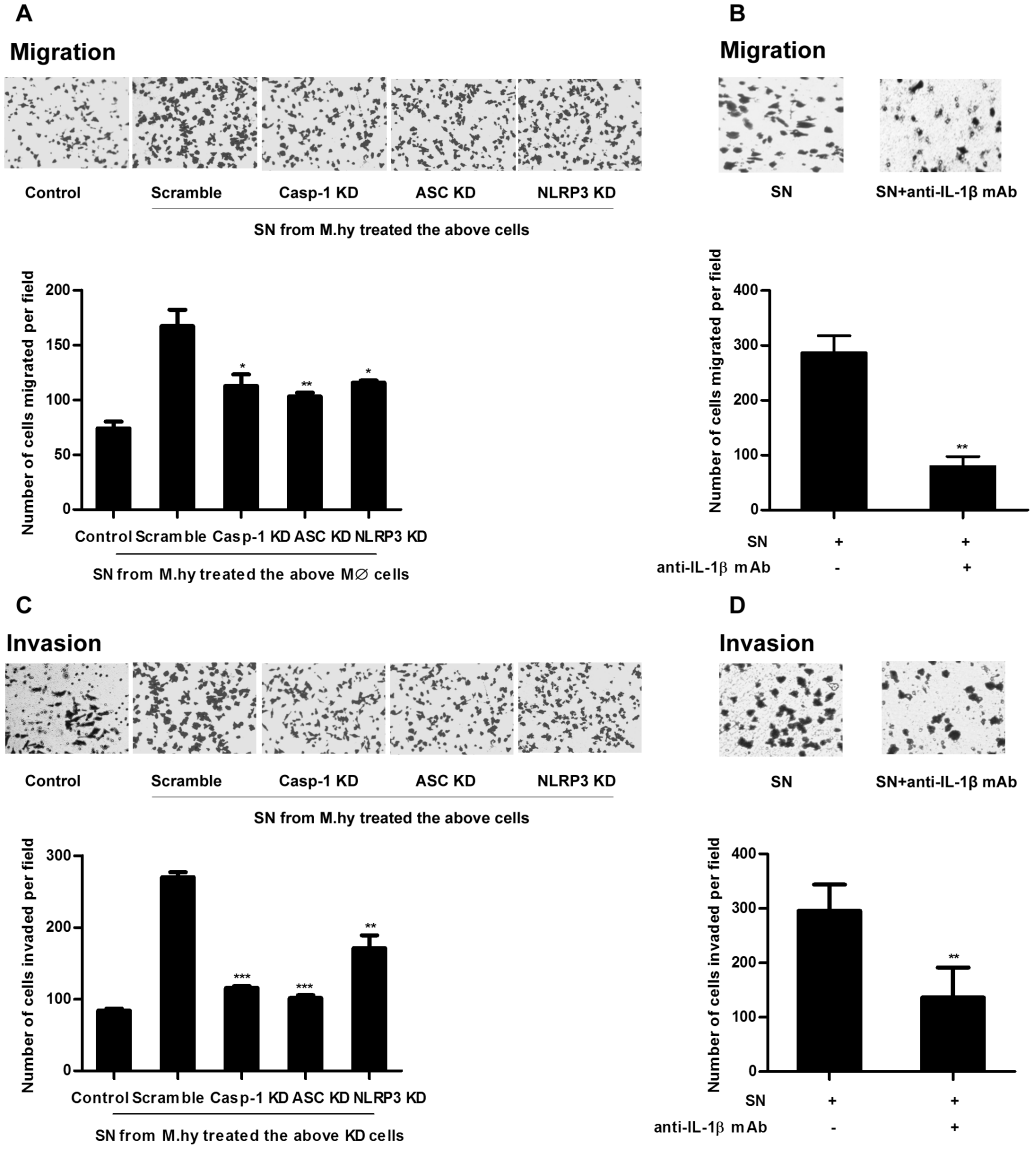
*M.hy* induced NLRP3 inflammasome activation promotes gastric cancer cell migration and invasion via IL-1β. A–B, 5×10^4^ MGC-803 cells treated with SNs from scramble macrophages as control, and SNs from *M.hy* treated Casp-1 KD, ASC KD and NLRP3 KD macrophages were analyzed by Transwell migration (A) and invasion (B) assays. C–D, 5×10^4^ MGC-803 cells treated with SNs from *M.hy* treated THP-1 derived macrophages or SNs containing anti-IL-1β mAb were analyzed by Transwell migration (C) and invasion (D) assays. The upper panels are migrated or invaded cells and the lower panels are average numbers of 4 microscopic fields for each corresponding experiments, respectively. Data are representative of three independent experiments and error bars represent SD. ***represents P<0.001, **represents P<0.01 and *represents P<0.05 in comparison with control in statistic analysis.

We next determined the effect of human recombinant IL-1β (rIL-1β) on gastric cancer cell carcinogenesis in a colony formation assay,which revealed that the rIL-1β did not influence the proliferation of gastric cancer cell line MGC-803 (Figure S7). Since IL-1β was reported to promote metastasis of other tumors [18], we checked the effects of rIL-1β on the migration and invasion of MGC-803 cells. Our results showed that the MGC-803 migration and invasion were enhanced with rIL-1β treatment (Figure 7A and 7C), while treatment with rIL-18 did not show any effect (Figure 7B and 7D). Next, we treated these gastric cancer cells with the culture supernatants from *M.hy* challenged THP-1 derived macrophages or macrophages with silencing of NLRP3, ASC and caspase-1 for migration and invasion assay. Our results showed that the culture supernatants from *M.hy* challenged scramble macrophage cells strongly enhanced migration and invasion of gastric cancer cells, while the culture supernatants from the NLRP3, ASC or caspase-1 knockdown macrophages did not, likely due to the diminished IL-1β secretion (Figure 8A and 8C). This enhanced migration and invasion of MGC-803 cells was abolished by treating the cells with anti-IL-1β mAb (Figure 8B and 8D). Taken together, our results demonstrated that *M.hy* induced migration and invasion of gastric cancer cell line MGC-803 by promoting IL-1β secretion from monocytic cells. Moreover, our data indicated that *M.hy* induced inflammasome activation may promote *M.hy* replication (Figure S8), which may create a positive feedback and finally leads to chronicity of disease.

## Discussion

Mycoplasmas distinguish themselves from other bacteria by small size, minute genome and lack of cell wall. Many mycoplasmas establish colonization and infection via adherence to host tissues. Because their dynamic surface architecture is antigenically and functionally versatile, mycoplasmas are capable of evading host immune attack and adapting to many habitats and cause chronic diseases [32]. Since the identification of *M.hy* from swine in 1962, little investigation was carried out on this pathogen till it was found to be associated with several human cancers [5], [41], [42], [43]. More recently, scientists found that *M.hy* protein p37 was responsible for promoting cancer cell invasiveness and metastasis [30], [44]. Besides this direct mechanism, this microbe may also induce tumorigenesis or metastasis indirectly through a local chronic inflammatory process. It’s well known that IL-1β is an important pro-inflammatory cytokine that promotes growth of colon cancer cells and enhances invasiveness and metastasis of B16 melanoma cells [19], [45]. Overexpression of IL-1β was found to induce gastric inflammation and cancer in mice [19]. In this study, we investigated whether the NLRP3 inflammasome, which controls IL-1β maturation, was activated by *M.hy*, and whether this activation can contribute to *M.hy* associated gastric metastasis. Our results showed that *M.hy* activated the NLRP3 inflammasome *in vitro* and *in vivo*, and the promotion of gastric cancer cell migration and invasion by *M.hy* was dependent on NLRP3 inflammasome activation. Besides NLRP3, we found that *M.hy* DNA also activated the AIM2 inflammasome, but MLAMP activation of NLRP3 was the dominant component for IL-1β induction by *M.hy.*

It was recently reported that acylated lipopeptide from heat-killed mycoplasma *Acholeplasma laidlawii* (HKAL) induced IL-1β production through NLRP7 inflammasome in human cells, while total HKAL induced IL-1β secretion was partially NLRP3 dependent [46], indicating that multiple inflammasomes can be activated by certain mycoplasma. Our data did not rule out the possible involvement of NLRP7 in *M.hy* induced IL-1β production, but *M.hy* mainly activated the NLRP3 inflammasome, because the IL-1β secretion was nearly completely abolished in the NLRP3 silenced cells (Figure 3H and 3I).

Early studies proposed that ROS activated the NLRP3 inflammasome by activating caspase-1 [47]. However, recent investigation showed that ROS inhibitors interfered with the transcription of IL-1β and NLRP3, but not affected activation of caspase-1 in mouse cells [40]. In our study, although ROS inhibition with low dose DPI (10 µM) significantly reduced the IL-1β production upon *M.hy* challenge, it did not inhibit the caspase-1 cleavage, the marker of inflammasome activation. However, when high dose of DPI (100 µM) was applied, caspase-1 cleavage was clearly inhibited (Figure 5E), suggesting that high dose of DPI can suppress the assembly of inflammasome complex, although we cannot exclude a possibility that this effect might have been resulted from the NLRP3 mRNA abolishing from baseline (Figure 5D). It is likely that the ROS inhibitor might interfere with both pro-IL-1β synthesis and caspase-1 activation.

How *M.hy* promotes ROS production in macrophage remains elusive. The lack of cell wall from *M.hy* permits direct contact of the mycoplasma membrane and host cell membrane, and this contact may lead to fusion of mycoplasma with eukaryotic host cell. This fusion may not only lead to mycoplasma components delivering into the host cell, but also allow insertion of the mycoplasma membrane components into the membrane of the eukaryotic host cell. Recently, *M.hy* membranes were found to possess Phospholipase A (PLA) that may be involved in the plasma membrane disruption process that occurs upon the invasion of host cells [48], [49]. During this process, the host cells may produce ROS. It would be worth of investigating whether PLA is required for the *M.hy* induced ROS production and IL-1β secretion.

As evidenced by our data, lysosomal rupture, K^+^ efflux, Ca^2+^ influx and ROS were all involved in *M.hy* induced NLRP3 activation. As reported before, K^+^ efflux and phagolysosomal rupture may induce Ca^2+^ influx, while Ca^2+^ influx may cause dysfunction of mitochondria, which results in release of oxidized mitochondrial DNA (mtDNA) into the cytosol, where it bind to and activate the NLRP3 inflammasome [9], [12]. Mitochondria seems to act as a central hub for integration of diverse signals sensed by NLRP3, but the relative contribution of ROS and mtDNA for NLRP3 inflammasome activation is still not clear to date [9].

Although *M.hy* is not highly virulent, it can establish chronic infection. By a gradual and progressive interaction with host cells, it induces chromosomal instability and malignant transformation, thus promoting tumor growth, migration or invasion [50], [51]. The *M.hy* p37 protein promoted gastric cancer cell, prostate carcinoma and melanoma cell lines invasiveness [30], [31]. In addition, the pro-inflammatory micro-environment such as IL-1β production may also contribute to this process [19]. Our study found that *M.hy* induced IL-1β promoted migration and invasion of the gastric cancer cells, while *M.hy* induced IL-18 did not contribute to the migration and invasion of gastric cancer cells, which was different from other reports [26], [27]. A possible explanation is that IL-18 may have promoted gastric cancer cells migration and invasion via the regulation of CD70, CD44 and VEGF expression in immune cells, which deserves our further investigation [26]. Another explanation could be that the dose of rIL-18 they used (150 ng/ml) is much higher than what we used [27]. Moreover, IL-1β promoted migration and invasion of the gastric cancer cells may be mediated by matrix metalloproteinase-9 (MMP9) as other searchers reported [52]. However, to get direct and more solid evidence and the corresponding mechanisms about the connection between NLRP3 inflammasome activation triggered by *M.hy,* and gastric cancer metastasis, appropriate animal models such as the *in vivo* tumor invasion assay [53] and patient studies are needed for further study in the future.

## Materials and Methods

### Cell Culture

THP-1 cells, PMA-induced macrophages and gastric cancer cell MGC-803 were maintained in RPMI 1640 medium with essential supplements. Human monocytes were isolated by Percol TM density-gradient centrifugation (G.E Healthcare, Bio-sciences, Sweden) from Human peripheral blood mononuclear cells (PBMCs) obtained from Shanghai Blood Center (Shanghai, China).

### *M.hy* Strain and PCR Detection

*M.hy* strain used in our study was obtained from contaminated cell culture. Mycoplasm detection was carried out through two rounds of PCR with cell culture medium [54]: First, run PCR with four primers 5′-ACACCATGGGAGYTGGTAAT-3′, 5′-CTTCWTCGACTTYCAGACCCAAGGCAT-3′, 5′-AAAGTGGGCAATACCCAC GC-3′, 5′-TCACGCTTAGATGCTTTCAGCG-3′ with cell cultured medium as template. Second, take 1 µl of the above PCR product as template and run PCR with three primers 5′-GTGSGGMTGGATCACCTCCT-3′, 5′-GCATCCACCAWAWACY CTTT-3′, 5′-CCACTGTGTGCCCTTTGTTCCT-3′. The first and second PCR runs under the same cycle condition. Then we sequenced the PCR product and obtained the following sequences: ACTCTTACTTAATTTAAAAGTTAATACAACTTTAATATT GCCTATTATTGCTAAAGATAAATATCTTAAGGTATTTTAATATTGGTAATCTATTTTAGAAATTTAATTTAAAAATTAAACTCGGTTATAAAAAAGATCGTTGAAATAATAAATATGAAGTTAATCATATTGTTATTTGCTATTCAGTTTTCAAAGAACTATAATTGAGAACTTAAAGCTCTCAAAACTAGACACGAATCGATTATGTAATAAGTCAATTAAGACTAACGGAAAGCGGAAAAAGAAGGTGATCCGTCCCCACG. By BLAST in NCBI database, we obtained the information that the mycoplasma is *M.hy*. It may be SK76, GDL-1 or MCLD strain.

### *M.hy* Culture, DNA Extraction and MLAMP Preparation

*M.hy* was cultivated in modified SP-4 medium containing 20% new-born bovine serum, 10% yeast extract, 1% glucose, 0.00024% phenolsulfonphthalein, and 1000 IU/ml penicillin. DNA extraction from *M.hy* was conducted according to the instructions with the reagents provided by Shanghai Lifefeng Biotechnology Co., Ltd. *M.hy* was quantified as color change units (CCU) per milliliter as described [55]. The extraction of MLAMP was carried out as described previously [34].

### Real-time PCR

NLRP3 and IL-1β mRNA expression were determined by Quantitative real-time PCR. Relative quantification of genes were normalized against an endogenous control β-actin via formula [2^–ΔCt(target gene-β-actin)^]. The primers used were: Homo sapiens (hs) IL-1β, 5′-CACGATGCACCTGTACGATCA-(forward)3′, 5′-GTTGCTCCATATCC TGTCCCT-(reverse)3′; β-actin, 5′-AGTGTGACGTGGACATCCGCAAAG-(forward)3′, 5′-ATCCACATCTGCTGGAAGGTGGAC-(reverse)3′; NLRP3, 5′-AAGGGCCATGGACTATTTCC-(forward)3′, 5′-GACTCCACCCGATGACAGTT-(reverse)3′. *M.hy* DNA copies were dertermined by Quantitative real-time PCR with standard-curve methodology for absolute copy numbers. The specific primers for *M.hy* were: 5′- CGATTCGTGTCTAGTTTTGAG -(forward)3′, 5′- ATTGCCTATTATTGCTAAAG -(reverse)3′.

### ELISA

Supernatants from cultured cells, mouse serum and mouse peritoneal lavage fluid were analyzed for cytokines IL-1β, IL-18, IL-6 or IL-8 secretion by ELISA (BD Biosciences) according to the manufacturer’s instructions.

### Generation of THP-1 Cells Expressing shRNAs Targeting Genes of Interest

shRNA vectors against human NLRP3, caspase-1 and ASC, and their scramble vectors are gifts from Dr. Jurg Tschopp [56]. About the generation of THP-1 cells expressing shRNAs, briefly, nt GATGCGGAAGCTCTTCAGTTTCA of human ASC coding sequence, nt CAGGTTTGACTATCTGTTCT of human NLRP3 coding sequence, nt GTGAAGAGATCCTTCTGTA of the 3′ UTR of human caspase-1 were inserted into pSUPER. The Pol III promoter shRNA cassettes from these vectors and from a lamin A/C-specific pSUPER control construct were inserted into the lentiviral vector pAB286.1, a derivative of pHR that contains a SV40-puromycin acetyl transferase cassette for antibiotic selection. Second-generation packaging plasmids pMD2-VSVG and pCMV-R8.91 were used for lentivirus production.

### Inactivation of *M.hy*

For heat inactivation, *M.hy* (10^6^ CCU/ml) was incubated at 60°C for 30 minutes. UV-inactivated *M.hy* was achieved by subjecting the pelleted *M.hy* to ultra-violet irradiation for 30 minutes. No growth was observed by the inoculation of heat and UV-inactivated *M.hy* during a 2-week period in modified SP-4 medium.

### Immunoblotting

For immunoblotting, cells were lysed in buffer containing 10 mM Tris (pH 7.5), 1% NP-40, 150 mM NaCl, and protease inhibitor cocktail. Proteins were separated on SDS-PAGE and then transferred onto PVDF membranes. The membranes were blocked with 5% fat free milk in 1×TBS with 0.05% Tween-20 and then probed with primary antibodies as follow: rabbit anti-human IL-1β (D116, Cell Signaling, USA), caspase-1 (sc-515, Santa Cruz, USA) and ASC (SC-22514-R, Santa Cruz, USA), goat anti-human pro-IL-1β (sc-1250, Santa Cruz, USA), mouse anti-human NLRP3 (ALX-804-881, Enzo Life Sciences, USA) and β-actin (KM9001, Tianjin Sungene Biotech, China). Appropriate HRP-conjugated secondary antibodies were used for signal detection via ECL reagent (Amersham, USA).

### ASC Pyroptosome Detection

ASC oligomerization assay was performed as described before [57]. Briefly, cytosolic lysates from *M.hy*, LPS and MSU treated cells were enriched for fractions by low-speed centrifugation and subjected to cross-linking with disuccinimidyl suberate (2 mM). The crosslinked samples were analyzed for ASC by immunoblotting.

### ROS Detection

Intracellular ROS was measured with the ROS-specific fluorescent probe CM-H2DCFDA (Molecular Probes, Invitrogen). THP-1 cells were loaded for 15 minutes with 2 µM CM-H2DCFDA, washed twice with PBS and then exposed to *M.hy* for different time points. The level of fluorescence was determined by flow cytometry.

### Cell Migration, Invasion and Clonogenic Assay

We performed cell migration and invasion assay using Transwell chamber with 8.0 µm polycarbonate membranes and or matrigel (BD Biosciences, USA) following a described method [58]. Anti-human IL-1β antibody was used for neutralization (eBioscience, USA). For clonogenic assay, MGC-803 cells were seeded at 500 cells/well in six-well plates, and treated with different concentrations of IL-1β for 7 days. Colonies were fixed and stained with 6% glutaraldehyde and 0.5% crystal violet.

### Animals

The generation of mice deficient in ASC and NLRP3 has been reported [59], [60], [61]. Caspase-1 deficient mice were purchased from the Jackson laboratory and backcrossed to C57BL/6 genetic background. C57BL/6 mice from Shanghai Laboratory Animal Center (SLAC) were used as wild-type control. All procedures complied with national guidelines and were approved by the Animal Care and Use Committee at Institut Pasteur of Shanghai.

### *In vitro M.hy* Challenge

5×10^4^ THP-1 cells, PMA-induced macrophages or the inflammasome components silenced THP-1 cells and macrophages induced by PMA, human primary monocytes and mouse BMDCs (prepared as described) [62] were treated with *M.hy* at a concentration of 6.7×10^7^ CCU/ml. The supernatants were harvested in the indicated time points for determination of cytokines expression by ELISA or immunoblotting.

### *In vivo M.hy* Challenge

Animals were anesthetized and challenged by intraperitoneal injection of 5×10^8^ CCU/ml of *M.hy* in 200 µl PBS. Mice were observed for up to 24 hours post challenge. *M.hy*-inoculated mice were euthanized at 2, 6, 12 and 24 hours after challenge, serum and peritoneal lavage fluid (PLF) were collected for cytokines levels determination by ELISA.

### Statistical Analysis

Data were analyzed for statistical significance by two-tailed student’s t test. Differences in P values ≤0.05 were considered statistically significant.

## Supporting Information

Figure S1 ***M.hy*** **induced IL-8 and IL-6 secretion in macrophages.** PMA-induced macrophages were treated with 6.7×10^7^ CCU/ml *M.hy*, 1 ug/ml LPS was positive control. 12 hours later the SNs were harvested for (A) IL-8 and (B) IL-6 secretion detection. Data presented are mean ± SD of one representative out of three independent experiments. A, **, P = 0.00241; B, *, P = 0.01843.
(TIF)

Figure S2 **MLAMP is the main component responsible for NLRP3 inflammasome activation.** 5×10^4^ THP-1 cells (A), PMA-induced macrophages (B) or PMA-induced scramble and NLRP3 KD macrophages (C) were incubated with DNase and RNase treated MLAMP (D/R) or normal MLAMP (no D/R treatment) for 12 hours, the SNs were harvested for IL-1β ELISA. Control was PBS containing DNase and RNase and reaction buffer. THP-1 cells were transfected with random plasmid or DNase treated plasmid or ssRNA40 and RNase treated ssRNA40 to demonstrate that the DNase and RNase works. Data presented are mean ± SD of one representative out of two independent experiments. A, **, P = 3.69305E-05; B, *, P = 0.02918; C, *, P = 0.01171.
(TIF)

Figure S3 ***M.hy*** **induced IL-1β secretion partially depends on AIM2 inflammasome.** A, 5×10^4^ PMA-induced scramble, AIM2 KD and NLRP3 KD macrophages were treated with 6.7×10^7^ CCU/ml *M.hy* or transfected with 1 ug/ml DNA extracted from *M.hy*, 12 hours later SNs were harvested for IL-1β ELISA. B, AIM2 KD efficiency is shown (AIM2 gene expression is inhibited by 95%). Data presented are mean ± SD of one representative out of three independent experiments. A, *, P = 0.01272 (comparison between DNA transfected scramble and AIM2 KD cells); *, P = 0.02935 (comparison between *M.hy* treated scramble and AIM2 KD cells); **, P = 0.00286 (comparison between *M.hy* treated scramble and NLRP3 KD cells).
(TIF)

Figure S4 **MGC-803 cells are infected by** ***M.hy*** **.** MGC-803 cells were treated with 6.7×10^7^ CCU/ml *M.hy*, 1 day and 7 days later the *M.hy* from SNs and cells were harvested by 12 000 g centrifugation for 15 minutes. Then the harvested *M.hy* quantity was detected via real-time PCR. Data presented are mean ± SD of one representative out of two independent experiments. **, P = 2.42974E-05.
(TIF)

Figure S5 ***M.hy*** **promotes MGC-803 migration and invasion.** 5×10^4^ MGC-803 cells treated with *M.hy* or control were analyzed by transwell migration (A) and invasion (B) assays. The upper panels are migrated or invaded cells and the lower panels are average numbers of 4 microscopic fields for each experiment, respectively. Error bars represent SD. A, **, P = 0.00001; B, **, P = 0.00224. Data are representative of three independent experiments.
(TIF)

Figure S6 ***M.hy*** **does not activate inflammasome in MGC-803 cells.** MGC-803 cells were infected with different doses of *M.hy*, 24 hours later SNs were harvested for IL-1β ELISA. Data presented are mean ± SD of one representative out of two independent experiments.
(TIF)

Figure S7 **IL-1β has no effect on proliferation of MGC-803 cells.** A, MGC-803 cells were seeded at 500 cells/well in six-well plates, and treated with different concentrations of IL-1β for 7 days. Colonies were fixed and stained with 6% glutaraldehyde and 0.5% crystal violet. And number of colonies was calculated with counter software. Data presented are one representative out of three independent experiments. B, Gastric cell line MGC-803 were seeded at 500 cells/well in 96-well plates. After 24 hours, IL-1β was added and incubated. At different time points, 10 µl of CCK8 (Cell counting kit-8) solution in 90 µl phosphate buffered saline (PBS) was added. Plates were incubated for an additional 1–4 hours. The optical density for each well was measured using a microculture plate reader at a wavelength of 450 nm.
(TIF)

Figure S8 ***M.hy*** **induced inflammasome activation may promote** ***M.hy*** **replication.** MGC-803 cells (A) or NLRP3 KD, Casp-1 KD and control Scramble cells (B) were treated with 6.7×10^7^ CCU/ml *M.hy*, at the same time, 1 ng/ml IL-1β or IL-18 were administrated, 2 days later the *M.hy* from SNs and cells were harvested by 12 000 g centrifugation for 15 minutes. Then the harvested *M.hy* quantity was detected via real-time PCR. Data presented are mean ± SD of one representative out of two independent experiments. A, from left to right, *represents P = 0.01020, 0.04529 respectively; B, **, P = 0.00772.
(TIF)
