# Supplementary figures and images for: Mycoplasma hyorhinis Activates the NLRP3 Inflammasome and Promotes Migration and Invasion of Gastric Cancer Cells
Source: PLoS One. 2013 Nov 6;8(11):e77955. doi: 10.1371/journal.pone.0077955 (PMC3819327; doi:10.1371/journal.pone.0077955)

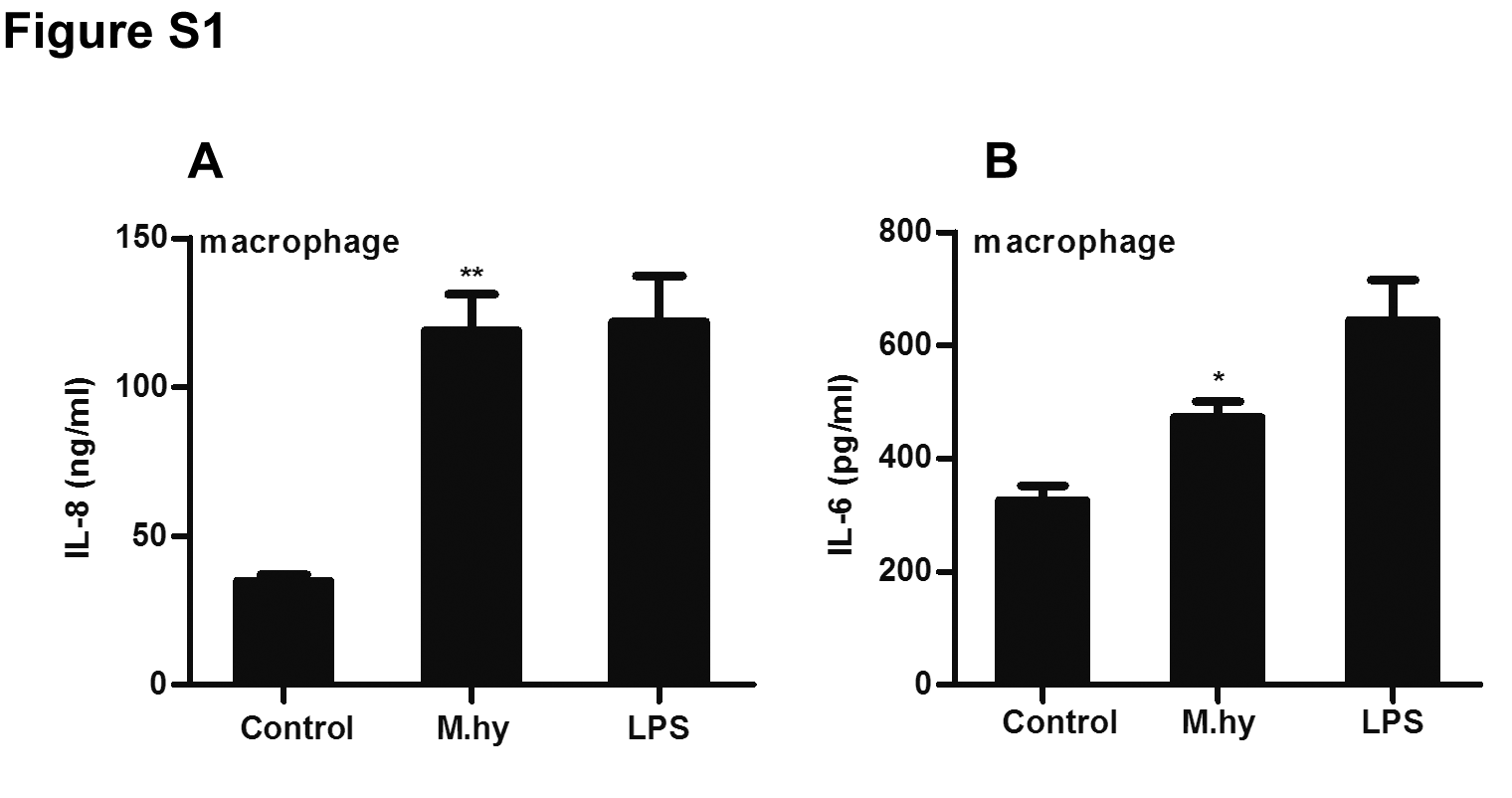

Supplement: Figure S1 — M.hy induced IL-8 and IL-6 secretion in macrophages. PMA-induced macrophages were treated with 6.7×10^7^ CCU/ml M.hy, 1 ug/ml LPS was positive control. 12 hours later the SNs were harvested for (A) IL-8 and (B) IL-6 secretion detection. Data presented are mean ± SD of one representative out of three independent experiments. A, **, P = 0.00241; B, *, P = 0.01843. (TIF) [file pone.0077955.s001.tif]

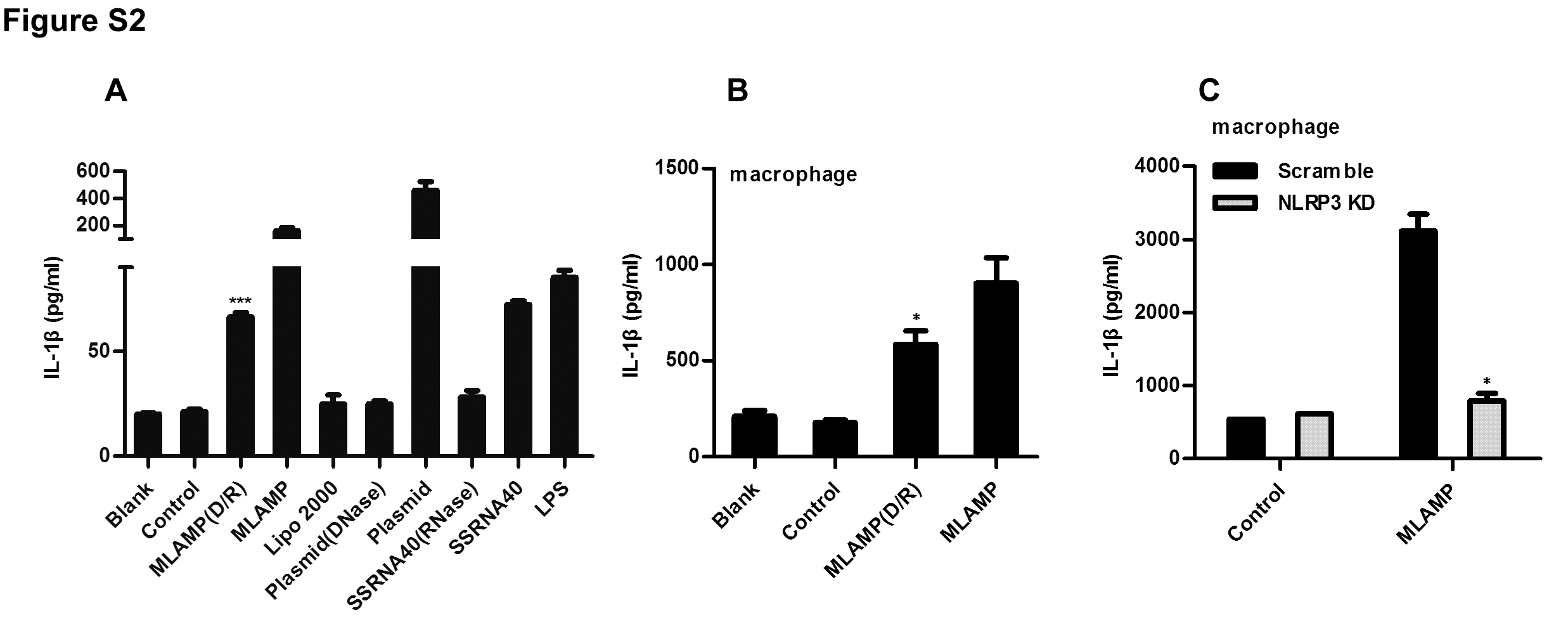

Supplement: Figure S2 — MLAMP is the main component responsible for NLRP3 inflammasome activation. 5×10^4^ THP-1 cells (A), PMA-induced macrophages (B) or PMA-induced scramble and NLRP3 KD macrophages (C) were incubated with DNase and RNase treated MLAMP (D/R) or normal MLAMP (no D/R treatment) for 12 hours, the SNs were harvested for IL-1β ELISA. Control was PBS containing DNase and RNase and reaction buffer. THP-1 cells were transfected with random plasmid or DNase treated plasmid or ssRNA40 and RNase treated ssRNA40 to demonstrate that the DNase and RNase works. Data presented are mean ± SD of one representative out of two independent experiments. A, **, P = 3.69305E-05; B, *, P = 0.02918; C, *, P = 0.01171. (TIF) [file pone.0077955.s002.tif]

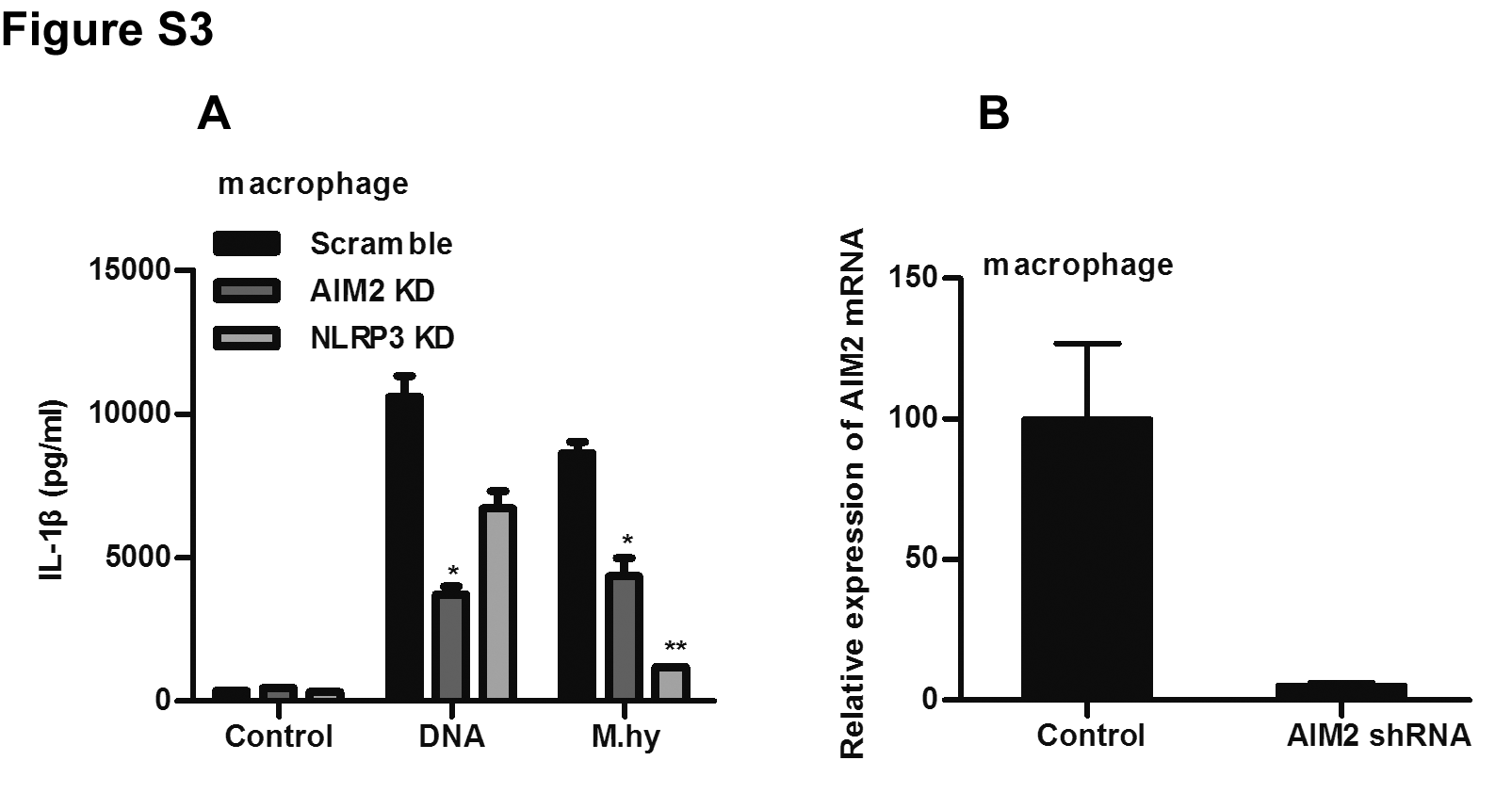

Supplement: Figure S3 — M.hy induced IL-1β secretion partially depends on AIM2 inflammasome. A, 5×10^4^ PMA-induced scramble, AIM2 KD and NLRP3 KD macrophages were treated with 6.7×10^7^ CCU/ml M.hy or transfected with 1 ug/ml DNA extracted from M.hy, 12 hours later SNs were harvested for IL-1β ELISA. B, AIM2 KD efficiency is shown (AIM2 gene expression is inhibited by 95%). Data presented are mean ± SD of one representative out of three independent experiments. A, *, P = 0.01272 (comparison between DNA transfected scramble and AIM2 KD cells); *, P = 0.02935 (comparison between M.hy treated scramble and AIM2 KD cells); **, P = 0.00286 (comparison between M.hy treated scramble and NLRP3 KD cells). (TIF) [file pone.0077955.s003.tif]

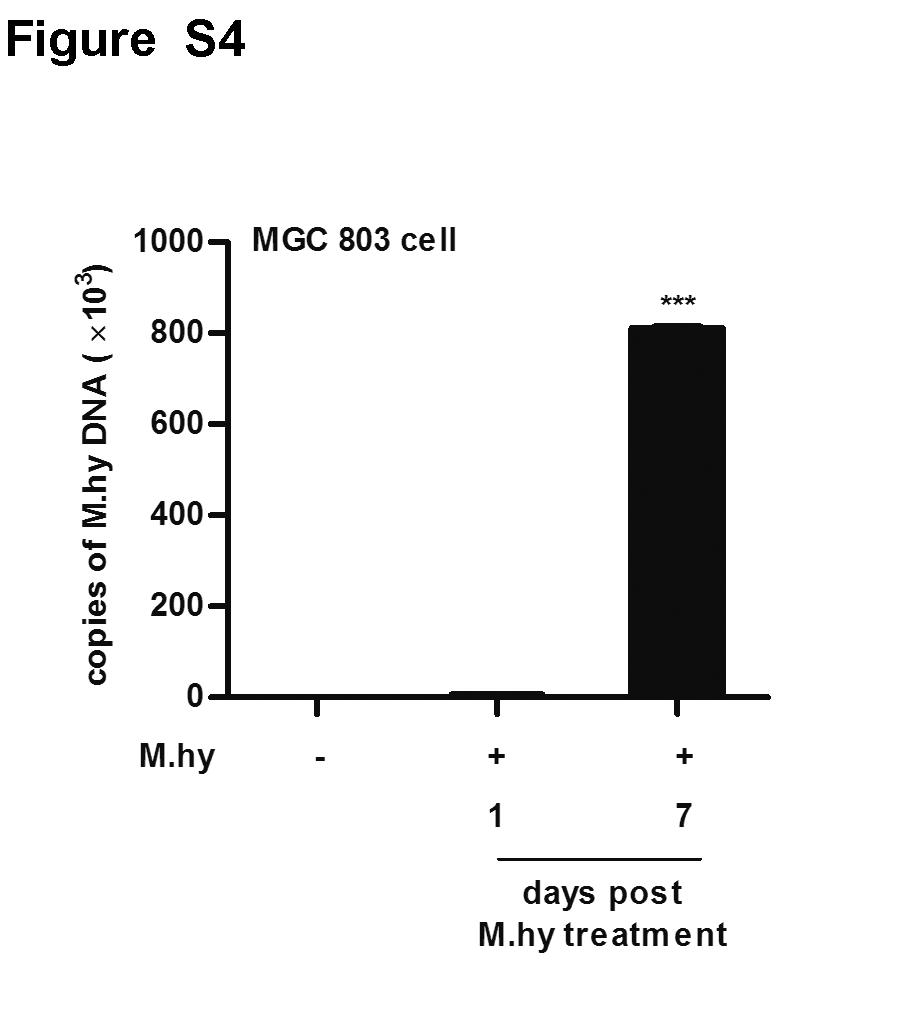

Supplement: Figure S4 — MGC-803 cells are infected by M.hy . MGC-803 cells were treated with 6.7×10^7^ CCU/ml M.hy, 1 day and 7 days later the M.hy from SNs and cells were harvested by 12 000 g centrifugation for 15 minutes. Then the harvested M.hy quantity was detected via real-time PCR. Data presented are mean ± SD of one representative out of two independent experiments. **, P = 2.42974E-05. (TIF) [file pone.0077955.s004.tif]

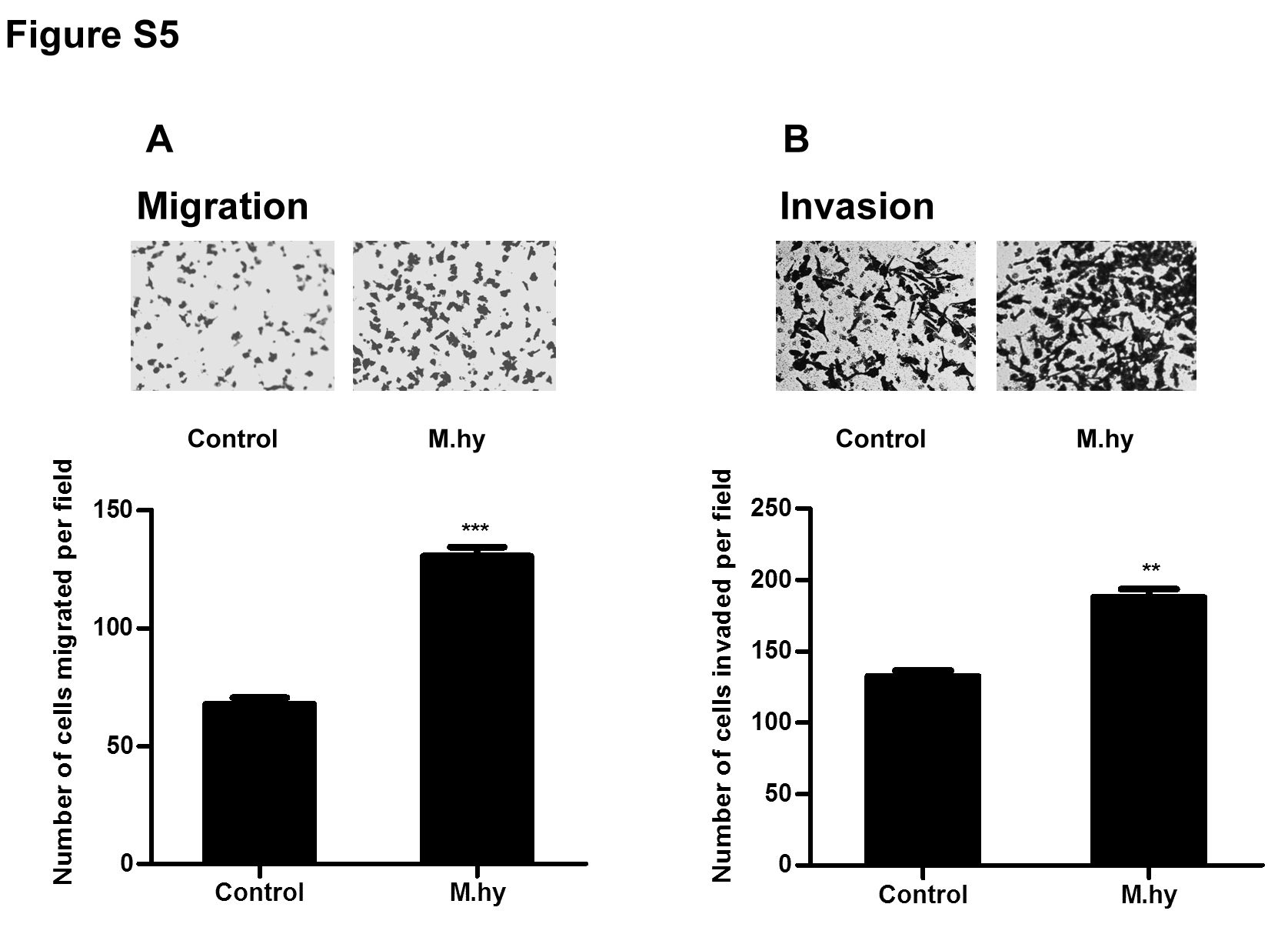

Supplement: Figure S5 — M.hy promotes MGC-803 migration and invasion. 5×10^4^ MGC-803 cells treated with M.hy or control were analyzed by transwell migration (A) and invasion (B) assays. The upper panels are migrated or invaded cells and the lower panels are average numbers of 4 microscopic fields for each experiment, respectively. Error bars represent SD. A, **, P = 0.00001; B, **, P = 0.00224. Data are representative of three independent experiments. (TIF) [file pone.0077955.s005.tif]

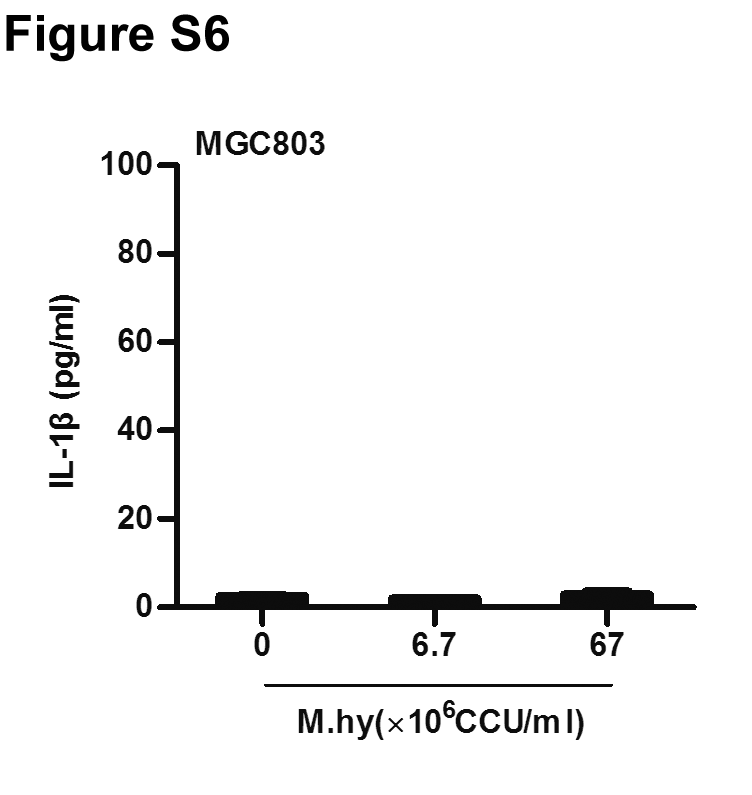

Supplement: Figure S6 — M.hy does not activate inflammasome in MGC-803 cells. MGC-803 cells were infected with different doses of M.hy, 24 hours later SNs were harvested for IL-1β ELISA. Data presented are mean ± SD of one representative out of two independent experiments. (TIF) [file pone.0077955.s006.tif]

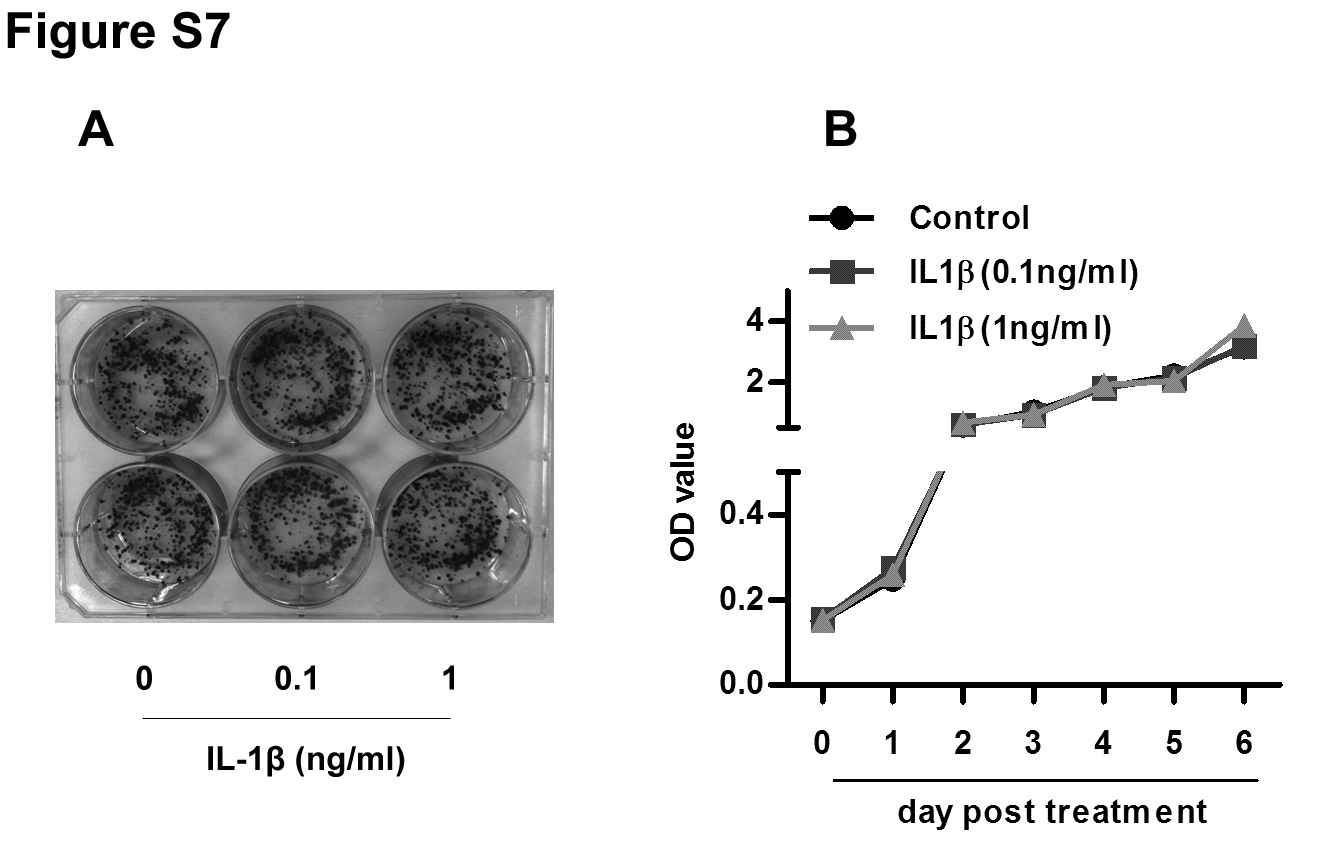

Supplement: Figure S7 — IL-1β has no effect on proliferation of MGC-803 cells. A, MGC-803 cells were seeded at 500 cells/well in six-well plates, and treated with different concentrations of IL-1β for 7 days. Colonies were fixed and stained with 6% glutaraldehyde and 0.5% crystal violet. And number of colonies was calculated with counter software. Data presented are one representative out of three independent experiments. B, Gastric cell line MGC-803 were seeded at 500 cells/well in 96-well plates. After 24 hours, IL-1β was added and incubated. At different time points, 10 µl of CCK8 (Cell counting kit-8) solution in 90 µl phosphate buffered saline (PBS) was added. Plates were incubated for an additional 1–4 hours. The optical density for each well was measured using a microculture plate reader at a wavelength of 450 nm. (TIF) [file pone.0077955.s007.tif]

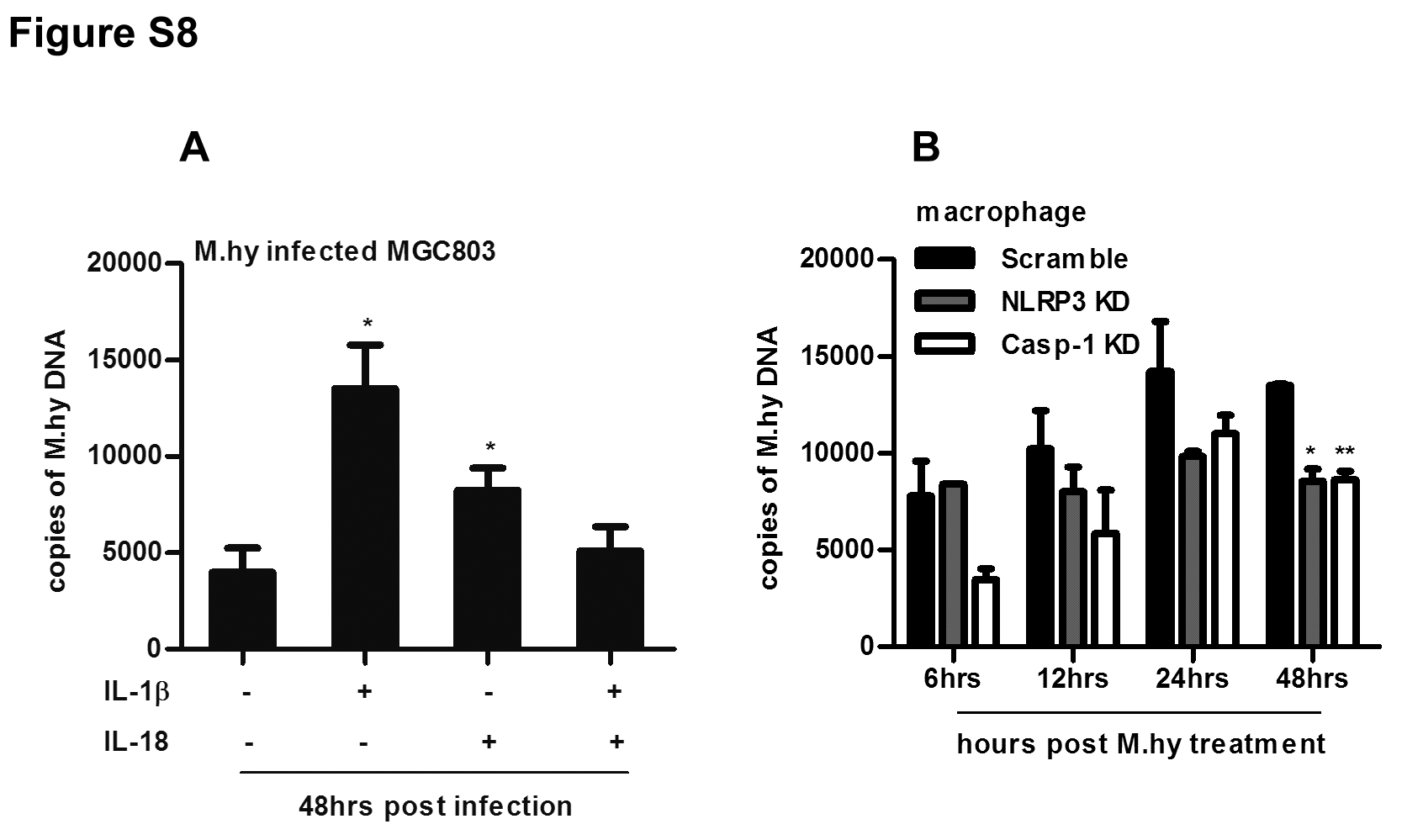

Supplement: Figure S8 — M.hy induced inflammasome activation may promote M.hy replication. MGC-803 cells (A) or NLRP3 KD, Casp-1 KD and control Scramble cells (B) were treated with 6.7×10^7^ CCU/ml M.hy, at the same time, 1 ng/ml IL-1β or IL-18 were administrated, 2 days later the M.hy from SNs and cells were harvested by 12 000 g centrifugation for 15 minutes. Then the harvested M.hy quantity was detected via real-time PCR. Data presented are mean ± SD of one representative out of two independent experiments. A, from left to right, *represents P = 0.01020, 0.04529 respectively; B, **, P = 0.00772. (TIF) [file pone.0077955.s008.tif]
